# Prevalence of dengue fever virus antibodies and associated risk factors among residents of El-Gadarif state, Sudan

**DOI:** 10.1186/s12889-018-5853-3

**Published:** 2018-07-27

**Authors:** Mawahib H. Eldigail, Gamal K. Adam, Rabie A. Babiker, Fatima Khalid, Ibrahim A. Adam, Osama H. Omer, Mohamed E. Ahmed, Sara L. Birair, Eltahir M. Haroun, Hassan AbuAisha, Abdelrahim E. Karrar, Hamid S. Abdalla, Imadeldin E. Aradaib

**Affiliations:** 1 Molecular Biology Laboratory (MBL), Department of Clinical Medicine, Faculty of Veterinary Medicine, University of Khartoum, P.O. Box 32, Khartoum North, Sudan; 2 Department of Medical laboratory Sciences, Faculty of Medicine, University of Elgadarif, Elgadarif, Sudan; 3 Department of Microbiology, Faculty of Science, University of Kassala, Kassala, Sudan; 4 Deanship of Scientific research, Alneelain University, Khartoum, Sudan; 5 Deanship of Scientific research, International University of Africa, Khartoum, Sudan

**Keywords:** Epidemiology, Survey, Dengue fever, ELISA, Sudan

## Abstract

**Background:**

Dengue fever, caused by dengue virus (DENV), has become one of the most important mosquito-borne viral diseases with a steady rise in global incidence, including the Sudan. Sporadic cases and frequent acute febrile illness outbreaks, compatible with Dengue fever, have been reported in El-Gadarif State, Sudan. However, diagnosis was based almost exclusively on clinical signs without confirmatory laboratory investigations. Despite the magnitude of the problem in El-Gadarif State, no information is currently available with regard to the epidemiology of the disease in this State. El-Gadarif State is one of the largest commercial centers in the Sudan. The objective of the present investigation is to estimate the prevalence of DENV antibodies, and determine the potential risk factors associated with seropositivity among residents of El-Gadarif State.

**Methods:**

A cross sectional study was conducted in a total of 701residents randomly selected from all 10 localities in El-Gadarif State. The sera from the 701 residents were tested for the presence of DENV-specific immunoglobulin G (IgG) antibodies using a commercially available Anti-dengue IgG enzyme-linked immunosorbent assay (ELISA).

**Results:**

Among the 701 residents, 334 residents (47.6%) were seropositive for DENV. Mosquito control (OR = 2.73, CI = 1.37–5.87, *p*-value = 0.001); low income (OR = 2.31, CI: 1.71–6.36, *p* value = 0.032); sleeping out-doors (OR = 3.73, CI = 2.63–6.23, *p*-value = 0.013), and localities were determined as potential risk factors for contracting DENV infection.

**Conclusions:**

The prevalence rate of DENV antibodies among residents of El-Gadarif State is significantly high (47.6%). Further epidemiologic studies including, distribution of mosquito vectors and implementation of improved surveillance are urgently warranted for better prediction and prevention of a possible DENV outbreak in El-Gadarif State, Sudan.

## Background

Dengue fever is a cosmopolitan mosquito-borne viral disease, and is the most important human arbovirus disease [1]. Dengue fever is caused by dengue virus (DENV), a member of the genus *Flavivirus* of the family *Flaviviridae*. The virus is transmitted by the infected female of the primary vector *Aedes aegypti* mosquitoes [2]. Infections can also be transmitted through blood transfusion, organ transplantation and possibly vertically from mother to child [3–7]. DENV infections often result in an acute, self-limiting, viral disease. However, the infection can be devastating and progresses to a fatal clinical disease characterized by substantial increase in vascular permeability resulting in shock [3]. Currently, DENV infection is increasingly recognized as one of the world’s emerging infectious diseases [8]. The detection of DENV-specific antibodies and circulation of the virus in infected people is well documented along the Red Sea coast of Eastern Sudan [9–13]. Recently, Kordufan region in western Sudan has witnessed several dengue fever outbreaks due to social unrest and war in the region [14, 15]. In the Sudan, the first outbreak of DENV was reported in 1986 among febrile patients in the Red Sea State, and DENV serotypes 1 and 2 were identified as the causative agents of the outbreak [16]. Serologic surveys have detected DENV antibodies in humans from different States of the Sudan, including Port Sudan, Kassala, Khartoum, and Kordufan [16–18]. A recent seroepidemiologic survey reported high prevalence of DENV-specific IgG among febrile patients in Kassala State in Eastern Sudan, suggesting significant circulation of DENV in the area, instead of being restricted to a particular point in the past [19, 20]. The recently reported high prevalence of DENV-specific antibodies in Kassala State was attributed to the constructed irrigated agricultural schemes and development of extensive urbanization. The presence of DENV-specific antibodies, and subsequent recovery of several DENV serotypes from infected patients have been reported in various regions of the Sudan [16–22]. The high prevalence of Dengue in the different states of the Sudan made its control a top priority. Epidemiologic studies, including implementation of improved surveillance, are extremely warranted and urgently needed for better prediction of the prevalence of this important arbovirus pathogen in the study area. The increasing incidence of dengue outbreaks in the Sudan warranted the current study to provide some information about the epidemiology of the disease in the study area. Dengue has become of great concern because of the frequent occurrence of sporadic cases and outbreaks among residents of urban areas [1, 3, 8]. Except for the DENV studies conducted along the Red Sea boarders, very little information is available about the epidemiology and disease potential in the Sudan. The current investigation was conducted to elucidate knowledge on the epidemiology of the disease, and determine the risk factors associated with the prevalence of DENV seropositivity among residents of El-Gadarif, one of the largest commercial State in the Sudan.

The objective of the present study is to estimate the prevalence of dengue fever by the detection of DENV-specific Ig G antibodies, and determine the potential risk factors associated with the infection with the DENV arbovirus among residents of El-Gadarif State, Sudan. It is quite probable that this study would assist in reducing the impact of infection on the livelihood of urban communities and prevent a possible DENV outbreak of the disease among residents in the study area of El-Gadarif State, eastern Sudan.

## Methods

### Study area

**Fig. 1:**
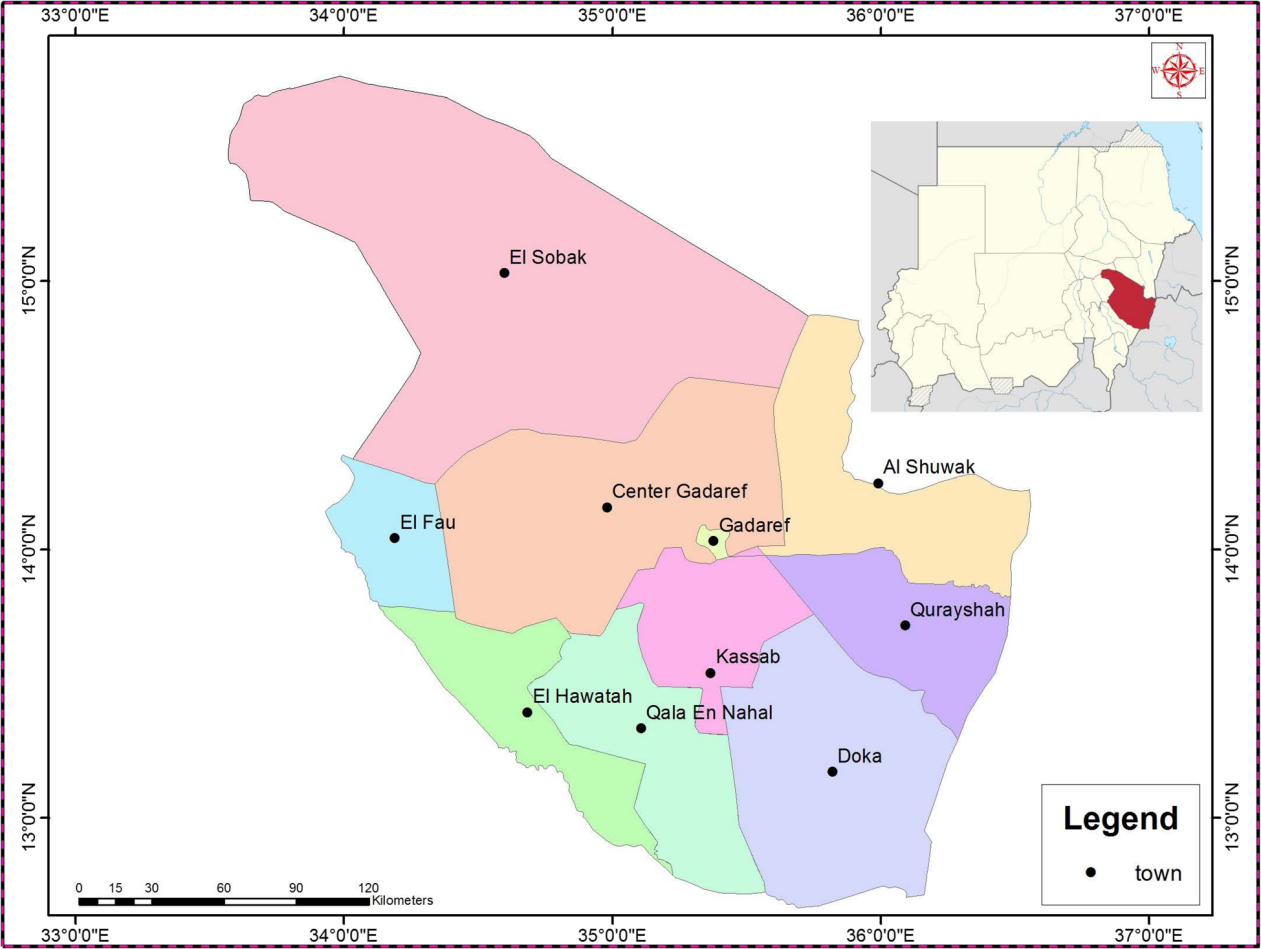
A map showing the localities included in the study area of El-Gadarif State, Sudan

This community-based, cross-sectional study was conducted in the urban area of El-Gadarif, one of the largest commercial States of the Sudan, during the period between August, 2016 and May, 2017. The state is characterized by vast areas of landscape suitable for agriculture, mainly depending on rainfall irrigation. The state is a strategic center that has the largest market for sesame and sorghum crops, and is considered as one of the pillars for food security, and a source of national economy in the Sudan; being the second largest commercial State in the country. El-Gadarif has a total population of 1,400,000 and covers an area of approximately 75, 263 km^2^. The State is located between latitudes 14° 02° - 5°.75° N and longitudes 35° 36–23° 0.38 E in the semi-desert tropics. The climate is hot and rainy in summer and cold in winter. The rainy season extends for 4 months, with an average annual rainfall of 700 to 900 mm. In the rainy season, large pools of water and green meadows with various types of acacia trees cover the land. The average temperature ranges from 21 °C in winter and 47 °C in summer. The mean annual evaporation rate is 7.7 mm/day, and the average relative humidity ranges from 21 to 44%. The localities included in the current investigation in El-Gadarif State are shown in Fig. 1.

### Method of the study

A cross sectional study was conducted to estimate the prevalence rate of DENV-specific IgG antibodies and determine the potential risk factors associated with the disease. As shown in Fig. 1 - El-Gadarif State has 10 localities, which include; Al-Showak (Al-Fashaga), El-Fau (El-Fau), Center Gadaref, El-Sobak (Butana), (Gadaref) El-Gadarif City,El-Hawatah (Al- Rahad), Kassab (Al-Gadaref Western Locality, Gala El-Nahal (Gala El-Nahal), Doka (Bassunda), and Al-Qurayshah locality (Al-Qurayshah).

A total of 701randomly selected participants from all 10 localities were included in the current investigation using the multistage probability sampling method. Briefly, all localities of Elgadarif state were considered in the study. Tow administration units were selected randomly from each locality. Five villages were selected from each administrative unit, which accounts for 10 villages per locality. Finally, 70 participants were selected from each of the 10 villages using simple random sampling. Thus, 700 samples were collected from the 10 localities of Elgadarif State. However, it should be noted that an additional blood sample was collected from Elfashaga locality, which resulted in a total number of 701 blood samples. All samples were collected using simple random sampling method. The participants included in this study were adults of over 18 years old.

### Study inclusion and exclusion criteria

We included in the study any individual aged ≥5 years old. The participants were members of a randomly selected household and residents in Elgadarif State, with or without symptoms. We excluded from the study those who have history of travailing to endemic area within last 2 weeks.

### Sample size

The prevalence of Dengue fever in Elgadarif State is unknown, therefore we assumed the.

seroprevalence of dengue antibodies to be 50%. The required sample size at 95% confidence and 50% prevalence and 0.04 absolute precision was calculated to be 600. A design effect of 2 and a non-response rate of 10% were considered to adjust for the sampling technique. The formula for the calculation of the sample size was applied as described by Martin et al., [23].

### Ethics approval and consent to participate

The ethical clearance for this study was obtained from the Ethics committee, Deanship of Scientific Research, Al-Neelain University, Sudan. All participants were provided with a written consent for the purpose of this study. The study objectives were explained to all participants before obtaining the informed consent. The structured questionnaire was employed to collect the risk factor information.

### Questionnaire

The collection of required data was made possible using a structured questionnaire through. Interviews were conducted for all participants from all above mentioned 10 localities. Interviewers were trained before conducting the survey to ensure that the questionnaires were well understood by the surveyors, avoiding the differences in the definitions and interpretations of the concepts used. All participants included in this study responded to the questionnaire, which covers socio-demographic characteristics including; age (young age, ≥ 5 and < 18 years-old; old age > 18); gender (male and female); disease awareness (yes, no); work (employed, unemployed); education (illiterate, primary, secondary, University); household income which includes, low income (less than 1,800 Sudanese Ginah (SDG), medium income (more than 1800 but less than 3000 SDG) and high income (more than 3000 SDG); presence of clean water containers (yes, no), mosquito vector (present or absent), mosquito control (practiced or not practiced).

### Laboratory investigations

Blood samples were collected from the participants, and sera were separated and kept frozen at − 20^0^ C until used for the detection of DENV-specific IgG antibodies by the enzyme-Linked Immunosorbent Assay (ELISA). The ELISA assay was performed using a commercially available non structural protein 1 (NS1) DENV ELISA Kit (Euroimmun AG, Luebeck, Germany), in accordance with the manufacturer’s specifications. The test samples were considered positive if the optical density was ≥ 50% of the mean of the negative controls.

### Statistical analyses

Statistical package for social sciences (SPSS) software package for window (version 21.0) was employed to enter the data in the computer. The univariable analysis using Chi-square (χ2) test was used to determine the associations between the outcome variable (status of dengue seropositivity) and its potential risk factors. Significant association between dengue fever and risk factor was initially considered when *P* value < 0.25 (two tailed; α = 0.25). The results of the univariable analysis were further subjected multivariable analysis using logistic regression. Results were expressed as odd ratios (OR) with 95% confidence intervals (C.I) for each risk factor. P value less than 0.05 (*p* < 0.05) represents significant association between the dengue fever seropositivity and associated risk factors [24].

## Results

DENV-specific IgG antibodies were detected in 334 out of 701 participants as determined by the competitive ELISA. This study recorded a prevalence rate of 47.6% for DENV antibodies among residents of El-Gadarif State. The highest and lowest rates of DENV seropositivity were 65.7 and 22.9%, respectively, which were recorded in Bassunda and Al-Qurayshah localities. Seven risk factors with *p*- value < 0.25 (two tailed; α = 0.25) were reported to be significantly associated with DENV-antibodies as calculated in the χ2 test. These risk factors included locality (*p*- value = 0.001), age (*p*- value = 0.123), gender (*p*- value = 0.145), income (*p*- value = 0.039), sleeping out-doors (*p*- value = 0.013), mosquito nets (*p*- value = 0.112), and mosquito control practice (*p*- value = 0.001). The results of the univariate analysis are presented in (Table 1). The significant results of univariate analysis were further analyzed in the final model of multivariate analysis using logistic regression to exclude confounding factors. The final model of DENV infection indicated that four potential independent risk factors were statistically significant. These potential risk factors included, mosquito control practice (OR = 2.73, CI = 1.37–5.87, *p*-value = 0.001); Low income (OR = 2.31, CI: 1.71–6.36, *p* value = 0.032); sleeping out-doors (OR = 3.73, CI = 2.63–6.23, *p*-value = 0.013), and localities. The six localities associated with high DENV serpositivity included Bassunda (OR = 4.09, CI: 1.35–6.91, *p* value = 0.007); Gadarif (OR = 2.42, CI: 1.94–4.99, *p*-value = 0.013), El-Rahad (OR = 2.51, CI = 1.61–5.54, *p*-value = 0.015); West Gallabat (OR = 2.13, CI = 1.05–4.31, *p*-value = 0.036); East Gallabat (OR = 2.73, CI = 1.30–5.71, *p*-value = 0.004); and El-Fashaga (OR = 3.27, CI = 1.58–6.76, *p*-value = 0.001). The significant association between DENV seropositivity and potential risk factors in the final model are shown in (Table 2). Age, gender, work, education, water containers, and mosquito vector were not significantly associated with DENV seropositivity.

**Table 1:** Univariate analysis for the association between potential risk factors and DENV infection among residents in El-Gadarif State, Sudan, using chi-square test

Risk factors	Cases tested	Cases affected (%)	df	*χ*2	*p*-value
Locality					
Gadaref	70	21 (30%)	9	59.5	0.001
Center Gagarif	70	37 (52.9%)			
Butana	70	19 (27.1%)			
E lfau	70	37 (52.9%)			
Al Rahad	70	41 (58%)			
Bassunda	70	46 (65.7%)			
West Galabat	70	34 (48.6%)			
East Galabat	70	39 (55.7%)			
Qurayshah	70	16 (22.9%)			
Elfashaga	71	44 (62%)			
Age					
young	176	91 (51.7%)	1	1.55	0.123
Old	525	243(46.3%)			
gender					
female	282	127(45%)	1	1.29	0.145
male	419	207(49.4%)			
Education					
illiterate	186	90(48.4%)	4	2.02	0.732
primary	154	75(48.7%)			
secondary	199	95(47.7%)			
university	107	45(42.1%)			
informal study	55	29(52.7%)			
Income					
low	489	245(50.1%)	2	6.4	0.039
medium	153	59(38.6%)			
high	59	30(50.8)			
Work					
unemployed	345	166(48.1%)	1	0.06	0.806
employed	356	168(47.2%)			
Disease awareness					
no	645	308(47.8%)	1	0.03	0.849
yes	56	26(46.4%)			
Sleeping outdoor					
no	324	138(42.6%)	1	6.16	0.013
yes	377	196(52%)			
Mosquito net use					
No	400	201(50.2%)	1	2.52	0.112
yes	301	133(44.2%)			
Presence of Clean water container					
No	31	12(38.7%)	1	1.03	0.308
Yes	670	322(48.1%)			
Mosquito control					
No	313	126(40.3%)	1	12.38	0.001
yes	388	208(53.6%)

**Table 2:** Multivariate analysis using logistic regression model for significant association (*p* > 0.05) between risk factors and DENV seropositivity among residents in El-Gadarif State, Sudan

Risk factors	OR	95%C I	*P*-Value
Mosquito control			
yes	Ref		
no	2.73	1.37–5.87	0.001
Sleeping out-doors			
Yes	Ref		
No	3.75	2.63–6.23	0.013
Income			
high	Ref		
low	1.61	1.71–6.36	0.032
Locality			
Elguresha	Ref		
Gadarif	2.42	1.94–4.9.9	0.013
Elrahad	2.51	1.16–5.54	0.015
Bassunda	4.09	1.35–6.61	0.007
West Galabat	2.13	1.05–4.31	0.036
East Glabat	2.73	1.30–5.71	0.004
Elfashaga	3.27	1.58–6.76	0.001

## Discussion

Dengue is a mosquito-borne virus is of high morbidity rate causing economic losses in many tropical and subtropical regions of the world [25–27]. Dengue virus (DENV) is rapidly spreading as the result of urbanization, climatic changes and increased human movements. DENV has emerged as the most common vector-borne viral infection in the current century. The virus occurs primarily in rural areas, but has recently become of urban distribution due to development of extensive urbanization in rural areas [28]. In addition, dengue was also reported in many African countries neighboring the Sudan including, Southern Sudan; Ethiopia, Eritrea, Uganda, Kenya, Democratic Republic of Congo, Egypt and Libya [29]. In recent years, the distribution and nature of DENV and other hemorrhagic fever viruses including, Rift Valley fever virus (RVFV) and Crimean Congo hemorrhagic fever virus (CCHFV) have changed substantially [30, 31]. In previous epidemiological surveys, high prevalence rates (71.7%) of DENV seropositivity were reported among residents of Kassala [11]. Other studies reported prevalence rates of 9.4% in the same state [9, 19]. In the Red Sea State of eastern Sudan, the prevalence rate was reported to be 12.8% among pregnant women [10]. In the present study, the prevalence of DENV-specific antibodies in residents of El-Gadarif State was estimated to be 47.6%, which is significantly high among the population of the state.

The high sero-prevalence rate could be attributed to the newly constructed irrigation projects and agricultural schemes in this state, which provides a suitable habitat for the survival of the adult and larval stages of *Aedes* vectors in this region. In addition, the heavy rainfall in this State was suggested as a contributory factor to high prevalence of the disease. The wide circulation of DENV in El-Gadarif State and the risks that these virus serotypes pose on the health and welfare of the residents of this State warrant an extensive and improved surveillance system for this arbovirus pathogen in the Sudan.

In the present study area (El-Gadarif State), DENV seropositivity increased among low-income participants. Low income residents are at 2 fold risk of contracting DENV infection compared to high income residents (OR = 2.31, CI: 1.71–6.36, *p* value = 0.032). The neighborhood in which people had a lower socioeconomic status (unemployment), had higher dengue prevalence rates, suggesting that the socio-economic status of the population is a potential risk factor for dengue prevalence. This could be attributed to the fact that unemployment may pose a lifestyle behavior that provides a suitable habitat for the breeding of the mosquito vector, thus increasing the risk of mosquito contact. Sleeping out-doors was also recorded as a potential risk factor for contracting DENV infection. Residents sleeping out-doors were at approximately 4 fold risk of becoming infected with DENV (OR = 3.73, CI = 2.63–6.23, *p*-value = 0.013), due to the increased contact and bites of the infected mosquito vector (*Aedes aegypti*) causing DENV infection. There was also association between DENV seropositivity and the six localities of El-Gadarif states including, Bassunda (OR = 4.09, CI: 1.35–6.91, *p* value = 0.007); Gadarif (OR = 2.42, CI: 1.94–4.99, *p*-value = 0.013), El-Rahad (OR = 2.51, CI = 1.61–5.54, *p*-value = 0.015); West Gallabat (OR = 2.13, CI = 1.05–4.31, *p*-value = 0.036); East Gallabat (OR = 2.73, CI = 1.30–5.71, *p*-value = 0.004); and El-Fashaga (OR = 3.27, CI = 1.58–6.76, *p*-value = 0.001). This is probably due to high rain fall in these localities, which provide suitable habitat for the mosquito vector, which transmits the disease. Residents of Bassunda locality are at 4 fold risk of becoming infected with DENV compared to other localities, suggesting increased endemicity of this locality with DENV infection. There was strong association between DENV seropositivity and routine mosquito control practice. Application of mosquito control measures, such as spraying with insecticides protects against DENV infection and decreased the DENV seropositivity by approximately 3 folds (OR = 2.73, CI = 1.37–5.87, *p*-value = 0.001). It is, therefore, recommended that routine application of insecticides or insect repellents should be considered for the prevention of DENV infection. It is worth mentioning that the development of a vaccine against DENV infection is in progress but has not yet been produced on commercial basis [32, 33]. Therefore, vector control by residual spraying of houses with insecticides, education and extension programs for public awareness about dengue and its prevention or reduction of its prevalence are considered as important components in the prevention of DENV infection. Monitoring and elimination of mosquito breeding sites is an important preventive measure that should also be considered. The results obtained in the current investigation are highly suggestive that during or before dengue epidemics; rigorous efforts are to be directed towards neighborhoods of lower socioeconomic status, and/or areas with large vegetation coverage around individual houses.

The risk assessment studies indicated that there was no significant association between DENV seropositivity and the rest of the risk factors including, work, education, water containers, mosquito vector (present or absent), included in the study. It is worth mentioning that gender has no significant difference for DENV seropositivity, and both sexes are equally susceptible to infection with DENV. Likewise, there was no significant difference related to age of the participants, suggesting that all ages are susceptible for DENV infection. Lack of sufficient awareness of dengue may have contributed significantly to the undermining of the danger of the disease by health care workers. In addition, dengue is not a reportable disease in the Sudan, and its surveillance and diagnosis are not widely or consistently implemented throughout the country. Moreover, reports for surveillance and other research activities pertaining to dengue in Africa are also limited and are not always available [29]. In the Sudan, acute febrile diseases of unknown etiology are very common, particularly in the urban areas, and malaria is usually considered to be the primary cause [34–37]. In Sudan and most African countries, dengue virus infection is not enlisted among the differential diagnosis of the acute febrile diseases. It is, therefore, recommended that the practicing physicians in rural hospitals and clinics of El-Gadarif State should consider DENV in their differential diagnosis in patients showing symptoms indicative of acute febrile illness or hemorrhagic fevers.

Limitation of the study: In the present study, important information regarding the prevalence and risk factors of the disease was made available for the first time in Elgadarif state. However, one of the limitations of the study is reflected by the fact that the participants were selected regardless of the presence of an active infection or clinical symptoms of the disease, which makes it difficult to differentiate between primary and secondary dengue infections. In addition, seropositivity was assessed by detecting dengue IgG antibody levels. It is well documented that detection of Ig G is useful in an epidemiological survey to identify previous or past infections. Therefore, detection of recent Dengue virus infection is likely to be missed by the described ELISA assay. Detection of recent infection necessitates the screening for Ig M antibodies. Moreover, Household density and screens in the window were not added in the model. The household density is always associated with poverty and low socioeconomic status, particularly in developing countries, such like Sudan, which could be considered as another social predictor for dengue infections. In this study, we have included socioeconomic status but household density and screens in the window were not included in the model. It is recommended that additional models such as house hold density and window screens should be included in a future study plan as they are expected to be potential risk factors for contracting dengue infections. Furthermore, virus isolation attempts and subsequent sequencing of the serotypes responsible for the secondary and severity of the infection have not been assessed. Further study should be conducted in the future to overcome this limitation of the study.

## Conclusions

In conclusion, the results obtained from the current study were indicative of circulation of DENV in El-Gadarif State, Sudan, and that the residents of the State are at risk of developing the disease. The prevalence of Dengue fever is significantly high (47.6%) among residents of this State. Mosquito control, socio-economic status (income), sleeping outdoors and localities were recorded as potential risk factors for contracting Dengue fever. However, outbreaks of dengue hemorrhagic fever have not yet been reported in this state of the Sudan. The specific DENV serotypes circulating in the area remain to be identified. Virus isolation attempts and subsequent molecular characterization studies (viral genome sequencing and phylogeny) are deemed necessary for tracing the movement of the virus in the region and to determine the genetic lineages of DENV serotypes circulating in Sudan. Surveillance for Dengue among residents of the State and the distribution of mosquito vectors should continue for better understanding of the epidemiology of the disease, and to provide public health authorities an opportunity for anticipation and preparation for a possible DENV outbreak in El-Gadarif State, Sudan.

## Abbreviations

CI: Confidence interval
DEN: Dengue
DEN-V: Dengue virus
ELISA: Enzyme-linked immunosorbent assay
HRP: Horse radish peroxidase
Ig G: Immunoglobulin G
OR: Odd ratio
RT-PCR: Reverse transcriptase polymerase chain reaction
Μl: Microliter

## References

[1] World Health Organization (2009). Dengue: guidelines for diagnosis, treatment, prevention and control.

[2] Gubler DJ (2002). Epidemic dengue/dengue hemorrhagic fever as a public health, social and economic problem in the 21^st^ century. Trends Microbiol.

[3] Wilder-Smith A, Chen LH, Massad E, Wilson ME (2009). Threat of dengue to blood safety in dengue-endemic countries. Emerg Infect Dis.

[4] Stramer SL (2015). The potential threat to blood transfusion safety of emerging infectious disease agents. Clin Adv Hematol Oncol.

[5] Stramer SL, Hollinger FB, Katz LM, Kleinman S, Metzel PS, Gregory KR (2009). Merging infectious disease agents and their potential threat to transfusion safety. Transfusion.

[6] Weerakkody RM, Palangasinghe DR, Dalpatadu KP, Rankothkumbura JP, Cassim MR, Karunanayake P (2014). Dengue fever in a liver-transplanted patient: a case report. J Med Case Rep.

[7] Wiwanitkit V (2010). Unusual mode of transmission of dengue. J Infect Dev Ctries.

[8] Bhatt S, Gething PW, Brady OJ, Messina JP, Farlow AW, Moyes CL (2013). The global distribution and burden of dengue. Nature.

[9] Soghaier MA, Himatt S, Osman KE, Okoued SI, Seidahmed OE, Beatty ME, Elmusharaf K, Khogali J, Shingrai NH, Elmangory MM (2015). Cross-sectional community-based study of the socio-demographic factors associated with the prevalence of dengue in the Eastern part of Sudan in 2011. BMC Public Health.

[10] Elduma AH, Osman WM (2014). Dengue and hepatitis E virus infection in pregnant women in Eastern Sudan, a challenge for diagnosis in an endemic area. Pan Afr Med J.

[11] Abdalla TM, Karsany MS, Ali AA (2015). Correlation of measles and dengue infection in Kassala, Eastern Sudan. J Med Virol.

[12] Seidahmed OM, Hassan SA, Soghaier MA, Siam HA, Ahmed FT, Elkarsany MM, Sulaiman SM. Spatial and temporal patterns of dengue transmission along a Red Sea coastline: a longitudinal entomological and serological survey in Port Sudan city. PLoS Negl Trop Dis. 2012;6, e1821 10.1371/journal.pntd.0001821 PMC3459851 23029582

[13] Seidahmed OM, Siam HA, Soghaier MA, Abubakr M, Osman HA, Abd Elrhman LS, Elmagbol B, Velayudhan R (2012). Dengue vector control and surveillance during a major outbreak in a coastal Red Sea area in Sudan. East Mediterr Health J.

[14] Soghaier MA, Mahmood SF, Pasha O, Azam SI, Karsani MM, Elmangory MM, Elmagboul BA, Okoued SI, Shareef SM, Khogali HS, Eltigai E (2014). Factors associated with dengue fever IgGsero-prevalence in South Kordofan state, Sudan,in 2012: reporting prevalence ratios. J Infect Public Health..

[15] Farnon EC, Gould LH, Griffith KS, Osman MS, Kholy AE, Brair ME, Panella AJ, Kosoy O, Laven JJ, Godsey MS, Perea W, Hayes EB (2010). Household-based sero-epidemiologic survey after a yellow fever epidemic, Sudan. Am J Trop Med Hyg.

[16] Hyams KC, Oldfield EC, Scott RM, Bourgeois AL, Gardiner H, Pazzaglia G, Moussa M, Saleh AS, Dawi OE, Daniell FD (1986). Evaluation of febrile patients in Port Sudan, Sudan: isolation of dengue virus. Am J Trop Med Hyg.

[17] McCarthy MC, Haberberger RL, Salib AW, Soliman BA, El-Tigani A, Khalid IO, Watts DM (1996). Evaluation of arthropod-borne viruses and other infectious disease pathogens as the causes of febrile illnesses in the Khartoum Province of Sudan. J Med Virol.

[18] Watts DM, el-Tigani A, Botros BA, Salib AW, Olson JG, McCarthy M, Ksiazek TG. Arthropod-borne viral infections associated with a fever outbreak in the Northern Province of Sudan. J Trop Med Hyg 1994; 97: 228–230. 8064945

[19] Himatt S, Osman KE, Okoued SI, Seidahmed OE, Beatty ME, Soghaier MA, Elmusharaf K (2015). Sero-prevalence of dengue infections in the Kassala state in the Eastern part of the Sudan in 2011. J Infect Public Health.

[20] Abdallah TM, Ali AA, Karsany MS, Adam I (2012). Epidemiology of dengue infections in Kassala, Eastern Sudan. J Med Virol.

[21] Adam I, Jumaa AM, Elbashir HM, Karsany MS (2010). Maternal and perinatal outcomes of dengue in PortSudan, Eastern Sudan. Virol J.

[22] Malik A, Earhart K, Mohareb E, Saad M, Saeed M, Ageep A, Soliman A (2011). Dengue hemorrhagic fever outbreak in children in Port Sudan. J Infect Public Health..

[23] Martin SW, Meek AH, Willeberg P (1987). Veterinary epidemiology: principles and methods.

[24] Dean AG, Sullivan KM, Soe MM. OpenEpi: open source epidemiologic statistics for public health, version 3.01. 2010. www.OpenEpi.com.

[25] Endy TP, Anderson KB, Nisalak A, Yoon IK, Green S, Rothman AL (2011). Determinants of inapparent and symptomatic dengue infection in a prospective study of primary school children in Kamphaeng Phet, Thailand. PLoS Negl Trop Dis.

[26] OhAinle M, Balmaseda A, Macalalad AR, Tellez Y, Zody MC, Saborio S (2011). Dynamics of dengue disease severity determined by the interplay between viral genetics and serotype-specific immunity. Sci Transl Med.

[27] Hairi F, Ong CH, Suhaimi A, Tsung TW, bin Anis Ahmad MA, Sundaraj C (2003). A knowledge, attitude and practices (KAP) study on dengue among selected rural communities in the Kuala Kangsar district. Asia Pac J Public Health.

[28] Van Kleef E, Bambrick H, Hales S. The geographic distribution of dengue fever and the potential influence of global climate change. TropIKA.net 2010.

[29] Amarasinghe A, Kuritsk JN, Letson GW, Margolis HS (2011). Dengue virus infection in Africa. Emerg Infect Dis.

[30] Aradaib IE, Erickson BR, Karsany MS, Khristova ML, Elageb RM, Khidir IE, Karrar AE, Nichol ST (2013). Rift Valley fever, Sudan. 2007-2010. Emerg Infect Dis.

[31] Hassan OA, Ahlm C, Sang R, Evander M (2011). The 2007 Rift Valley fever outbreak in Sudan. PLoS Negl Trop Dis.

[32] Halstead SB (2012). Dengue vaccine development: a 75% solution?. Lancet.

[33] Hadinegoro SR, Arredondo-García JL, Capeding MR, Deseda C, Chotpitayasunondh T, Dietze R (2015). Efficacy and long-term safety of a dengue vaccine in regions of endemic disease. N Engl J Med.

[34] Nashed NWJ, Olson JG, Tigani AE (1993). Isolation of Batai virus (Bunyaviridae, Bunyavirus) from the blood of suspected malaria patients in Sudan. Am J Trop Med Hyg.

[35] Aradaib IE, Erickson BR, Mustafa ME, Khristova ML, Saeed NS, Elageb RM, Nichol ST (2010). Nosocomial outbreak of Crimean-Congo hemorrhagic Fever,Sudan. Emerging Infect Dis.

[36] Aradaib IE, Erickson BR, Karsany ME, Khristova ML, Elageb RM, Mohamed MEH, Nichol ST (2011). Multiple Crimean-Congo hemorrhagic fevervirus strains are associated with disease outbreaks in Sudan, 2008-2009. PLoS Negl Trop Dis.

[37] Hassanain AM, Noureldien W, Karsany MS, Saeed el NS, Aradaib IE, Adam I (2010). Rift Valley Fever among febrile patients at New Halfa hospital, eastern Sudan. Virol J.

