# Correction to: Prevalence of dengue fever virus antibodies and associated risk factors among residents of El-Gadarif state, Sudan

**DOI:** 10.1186/s12889-022-13984-2

**Published:** 2023-03-24

**Authors:** Mawahib H. Eldigail, Gamal K. Adam, Rabie A. Babiker, Fatima Khalid, Ibrahim A. Adam, Osama H. Omer, Mohamed E. Ahmed, Sara L. Brair, Eltahir M. Haroun, Hassan AbuAisha, Abdelrahim E. Karrar, Hamid S. Abdalla, Imadeldin E. Aradaib

**Affiliations:** 1 Department of Clinical Medicine, Faculty of Veterinary Medicine, Molecular Biology Laboratory (MBL), University of Khartoum, P.O. Box 32, Khartoum North, Sudan; 2 Department of Medical laboratory Sciences, Faculty of Medicine, University of Elgadarif, Elgadarif, Sudan; 3 Department of Microbiology, Faculty of Science, University of Kassala, Kassala, Sudan; 4 Deanship of Scientific Research, Alneelain University, Khartoum, Sudan; 5 Deanship of Scientific Research, International University of Africa, Khartoum, Sudan

**Correction to: BMC Public Health 18, 921 (2018)**

**https://doi.org/10.1186/s12889-018-5853-3**

The original publication of this article contained an incorrect author name. The incorrect and correct name are shown below. The original article [1] has been updated.

**Author name**

- The incorrect author name is: Sara L. Birair
- The correct author name is: Sara L. Brair
